# Identifying modifiable factors associated with neuroimaging markers of brain health

**DOI:** 10.1111/cns.70057

**Published:** 2024-10-15

**Authors:** Liang‐Yu Huang, Yan Fu, Yi Zhang, He‐Ying Hu, Ling‐Zhi Ma, Yi‐Jun Ge, Yong‐Li Zhao, Ya‐Ru Zhang, Shi‐Dong Chen, Jian‐Feng Feng, Wei Cheng, Lan Tan, Jin‐Tai Yu

**Affiliations:** ^1^ Department of Neurology Qingdao Municipal Hospital, Qingdao University Qingdao China; ^2^ Department of Neurology and Institute of Neurology Huashan Hospital, State Key Laboratory of Medical Neurobiology and MOE Frontiers Center for Brain Science, Shanghai Medical College, Fudan University Shanghai China; ^3^ Institute of Science and Technology for Brain‐Inspired Intelligence, Fudan University Shanghai China; ^4^ Key Laboratory of Computational Neuroscience and Brain‐Inspired Intelligence (Fudan University), Ministry of Education Shanghai China; ^5^ Fudan ISTBI—ZJNU Algorithm Centre for Brain‐Inspired Intelligence Zhejiang Normal University Jinhua China; ^6^ MOE Frontiers Center for Brain Science Fudan University Shanghai China

**Keywords:** brain health, exposome‐wide association study, magnetic resonance imaging, UK biobank

## Abstract

**Aims:**

Brain structural alterations begin long before the presentation of brain disorders; therefore, we aimed to systematically investigate a wide range of influencing factors on neuroimaging markers of brain health.

**Methods:**

Utilizing data from 30,651 participants from the UK Biobank, we explored associations between 218 modifiable factors and neuroimaging markers of brain health. We conducted an exposome‐wide association study using the least absolute shrinkage and selection operator (LASSO) technique. Restricted cubic splines (RCS) were further employed to estimate potential nonlinear correlations. Weighted standardized scores for neuroimaging markers were computed based on the estimates for individual factors. Finally, stratum‐specific analyses were performed to examine differences in factors affecting brain health at different ages.

**Results:**

The identified factors related to neuroimaging markers of brain health fell into six domains, including systematic diseases, lifestyle factors, personality traits, social support, anthropometric indicators, and biochemical markers. The explained variance percentage of neuroimaging markers by weighted standardized scores ranged from 0.5% to 7%. Notably, associations between systematic diseases and neuroimaging markers were stronger in older individuals than in younger ones.

**Conclusion:**

This study identified a series of factors related to neuroimaging markers of brain health. Targeting the identified factors might help in formulating effective strategies for maintaining brain health.

## Graphical abstract

We summarized the variables associated with the neuroimaging markers of brain health across six primary exposome categories: systematic diseases, lifestyle factors, social supports, anthropometric indicators, personality traits, and biochemical markers. These results highlight the potential of integrating diverse of exposures to devise effective strategies for preserving brain health.

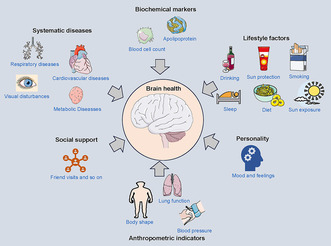

## 1 INTRODUCTION

Brain health is defined as the preservation of optimal brain structural integrity and normal brain function at a given age without obvious brain disorders that affect normal brain function. Structural alterations in the brain are well‐characterized markers of brain health. For instance, hippocampal atrophy serves as an early marker of dementia and cognitive decline.^1^ White matter hyperintensity (WMH) volume is an indicator of lesions in the white matter of the brain, which are common in the dementia subtypes of Alzheimer's disease (AD) and vascular dementia.^2^ Furthermore, decreased total brain, gray and white matter volumes have been reported in many brain disorders.^3^, ^4^ In the USA alone, in 2020, one in 10 elderly people (aged 65 years or older) had dementia; a stroke‐related death occurred every 4 min; and more than 20 million adults experienced at least one major depressive episode.^5^ Therefore, the brain health crisis demands a rapid response. Notably, brain structural alterations begin long before the presentation of brain disorder symptoms.^6^ Thus, identifying the modifiable factors influencing brain structural alterations could enable us to implement effective strategies for enhancing brain health.

Environmental and lifestyle risk factors can accelerate cellular and molecular aging, leading to faster aging phenotypes and earlier onset of chronic diseases.^7^ Cardiovascular risk factors and lifestyle factors have been linked to neuroimaging markers of brain health.^8^, ^9^, ^10^ However, traditional exposures representing only a small portion of the “exposome”—the cumulative exposure across an individual's lifetime.^11^ As a result, focusing on a limited number of exposures can lead to inflated effect sizes and type I errors. In contrast, the exposome‐wide association study (EWAS) offers a comprehensive approach, systematically evaluating multiple environmental factors and validating their associations with diseases.^12^ However, there have been very few EWAS focused on neuroimaging markers of brain health. Furthermore, even though the research explored the impact of various chronic diseases on brain volumes, it primarily focused on chronic diseases while overlooking the influence of lifestyle factors and other important exposures.^10^ Therefore, it is essential to address this gap by conducting comprehensive EWAS to explore the full range of modifiable factors that affect neuroimaging markers of brain health.

**FIGURE 1:**
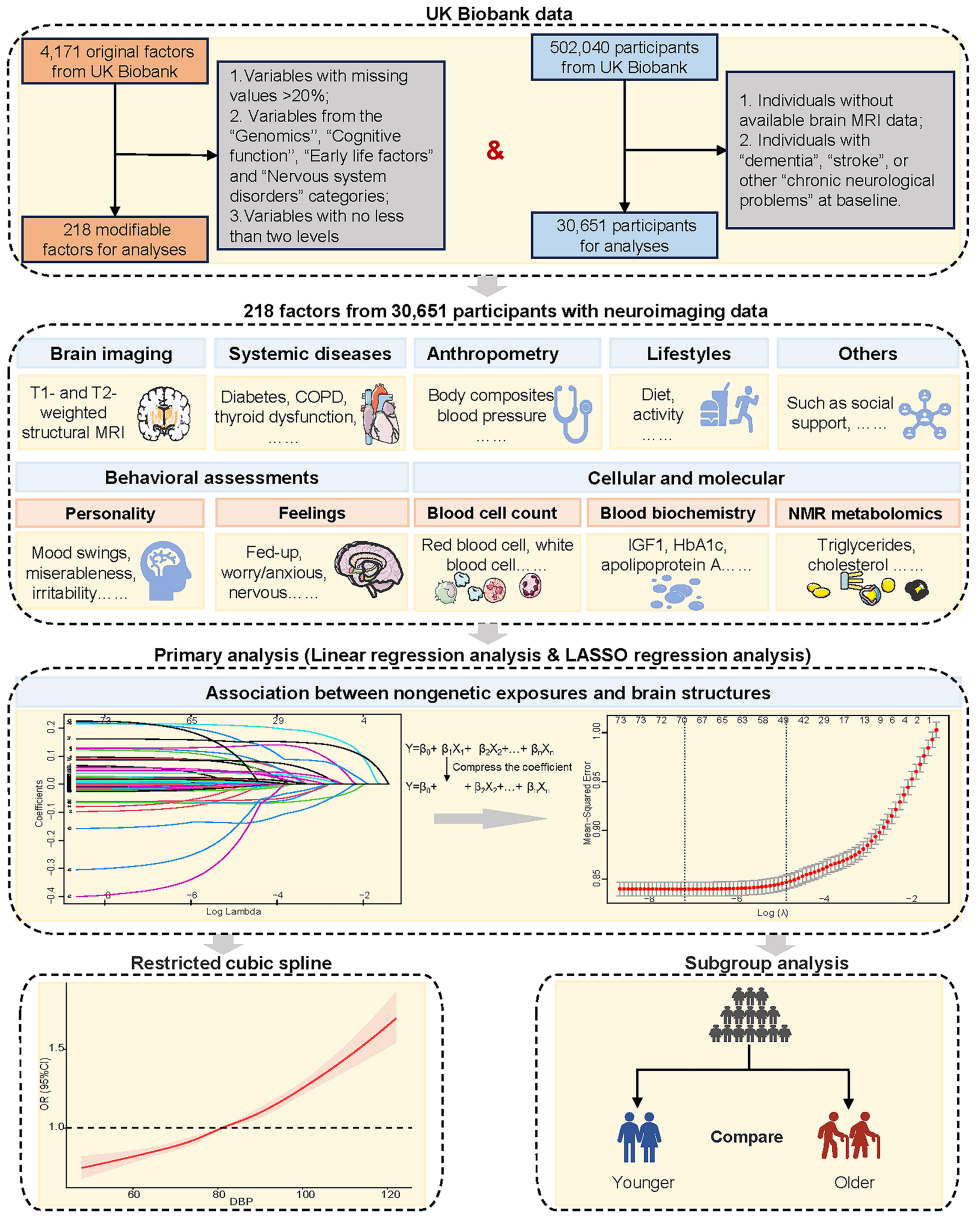
Overview of analytic design. Analytical procedure to identify modifiable risk factors associated with imaging markers of brain health in the UK Biobank. Abbreviations: LASSO, least absolute shrinkage and selection operator; COPD, chronic obstructive pulmonary disease; DBP, diastolic blood pressure; MRI, magnetic resonance imaging.

To achieve this, we employed the least absolute shrinkage and selection operator (LASSO) technique to conduct an exposome‐wide analysis, evaluating multiple factors in relation to neuroimaging markers of brain health, including WMH, total brain volume (TBV), hippocampal volume (HCV), gray matter volume (GMV), and white matter volume (WMV). In addition, restricted cubic splines (RCS) were used to estimate nonlinear correlations. Furthermore, stratified analyses were performed to explore potential heterogeneity (Figure 1).

## 2 METHODS

### 2.1 Participants

Data were obtained from individuals participating in UK Biobank (https://biobank.ndph.ox.ac.uk), a large‐scale longitudinal cohort that involved over 500,000 participants in 22 centers in the UK.^13^ Baseline demographic, physiological, and clinical data were gathered from March 2006 to October 2010, and the magnetic resonance imaging (MRI) data were gathered between August 2014 and October 2019. Only individuals with available brain MRI data and exposure information were included in the analysis. Individuals with dementia, Parkinson's disease, multiple sclerosis, epilepsy, stroke, or other chronic neurological conditions (nervous system infection, encephalitis, brain abscess, demyelinating disease, cerebral palsy, cerebral aneurysm, and brain hemorrhage) at baseline were excluded from the analysis (Figure 1; Figure S1). Informed consent was obtained from all participants at the time of recruitment.

### 2.2 Exposure variables

During the initial screening, we eliminated variables with more than 20% missing data and retained those that remained. Subsequently, we manually excluded unmodifiable exposure variables, including categories such as “Genomics,” “Cognitive Function,” “Early Life Factors,” and “Nervous System Disorders.” Next, we selected variables with at least two levels and treated any meaningless negative values as missing (NA). Ultimately, 218 modifiable variables were identified for further analysis. For binary variables, major diseases were identified using ICD‐10 codes and self‐reported disease data (as outlined in Table S1). For ordinal variables, we reordered levels to follow a logical progression (details in Table S2). For certain continuous variables, like hand grip strength, we created substitute variables by summing or averaging the values (see Table S3). All the variables were categorized into the following broad categories: (i) systemic diseases; (ii) lifestyle factors; (iii) personality traits and emotional factors; (iv) anthropometric indicators; (v) biochemical markers from blood tests; and (vi) other factors, including social support indicators.

### 2.3 Brain magnetic resonance imaging

We selected five brain imaging‐derived phenotypes as our outcomes, including TBV, GMV, WMV, WMH load, and HCV. Brain MRI data were captured by standard Siemens Skyra 3T running VD13A SP4, with a standard Siemens 32‐channel RF receive head coil.^14^ Then, the T1‐ and T2‐weighted scans underwent an automated pipeline based on the Functional MRI of the Brain Software Library. The general pipeline for the generation of imaging‐derived phenotypes has been described in detail elsewhere.^15^ TBV was computed by adding the GMV and WMV (excludes cerebrospinal fluid), while HCV was generated by averaging the left and right HCVs. Total and regional brain volumes were adjusted for head size based on the external surface of the skull, using the ratio‐corrected method. Due to the positively skewed distribution of WMH load, it was log‐transformed for the analysis.

### 2.4 Statistical analyses

Exposure variables with missing values less than 20% were imputed based on a maximum likelihood estimation method, guided by the observed correlation structure within the data. The Shapiro–Wilk tests were used to assess the normality of the data distribution, and dependent variables with skewed distributions were log‐transformed to achieve normalization. We employed Chi‐square tests for categorical variables and *t*‐tests for continuous variables to evaluate differences between groups in baseline characteristics. Extreme outliers for brain volume were excluded, and then standardized the data by converting them to *z*‐scores.

First, linear regression models were applied to test the associations between each exposure and neuroimaging markers, with a conservative Benjamini‐Hochberg procedure (false discovery rate [FDR] correction) corrected significance threshold for identifying potential factors. The significant factors were then incorporated into multivariable regression models using the LASSO technique to identify the top factors that influenced brain structures. The LASSO technique employs a regularization process that penalizes the coefficients of regression variables, causing some to shrink to zero. Second, to estimate the nonlinear relationships of exposures with neuroimaging markers, we utilized RCS with knots positioned at the 5th, 27.5th, 50th, 72.5th, and 95th percentiles of the exposures. Third, we derived weighted standardized scores for neuroimaging markers using the coefficients of each variable. A higher weighted standardized score represented poorer brain health. The amount of variance of neuroimaging markers accounted for by weighted standardized scores was computed by *R* ^2^ in regression models. Finally, we conducted subgroup analyses stratified by age at the time of the MRI examination (<65 years or ≥65 years) to test the potential modification effects of age on the associations between exposures and neuroimaging markers of brain health.

All analyses were controlled for age and sex. Statistical tests were conducted using R version 4.0.2 (The R Foundation for Statistical Computing, Vienna, Austria), with all *p*‐values being two‐sided and a significance threshold set at <0.05.

## 3 RESULTS

A total of 30,651 participants were included in our study (mean age: 64.34 years, standard deviation: 7.69 years; 50.94% women). Baseline demographic characteristics of the participants are shown in Table S4.

### 3.1 Exposome‐wide association analyses

**FIGURE 2:**
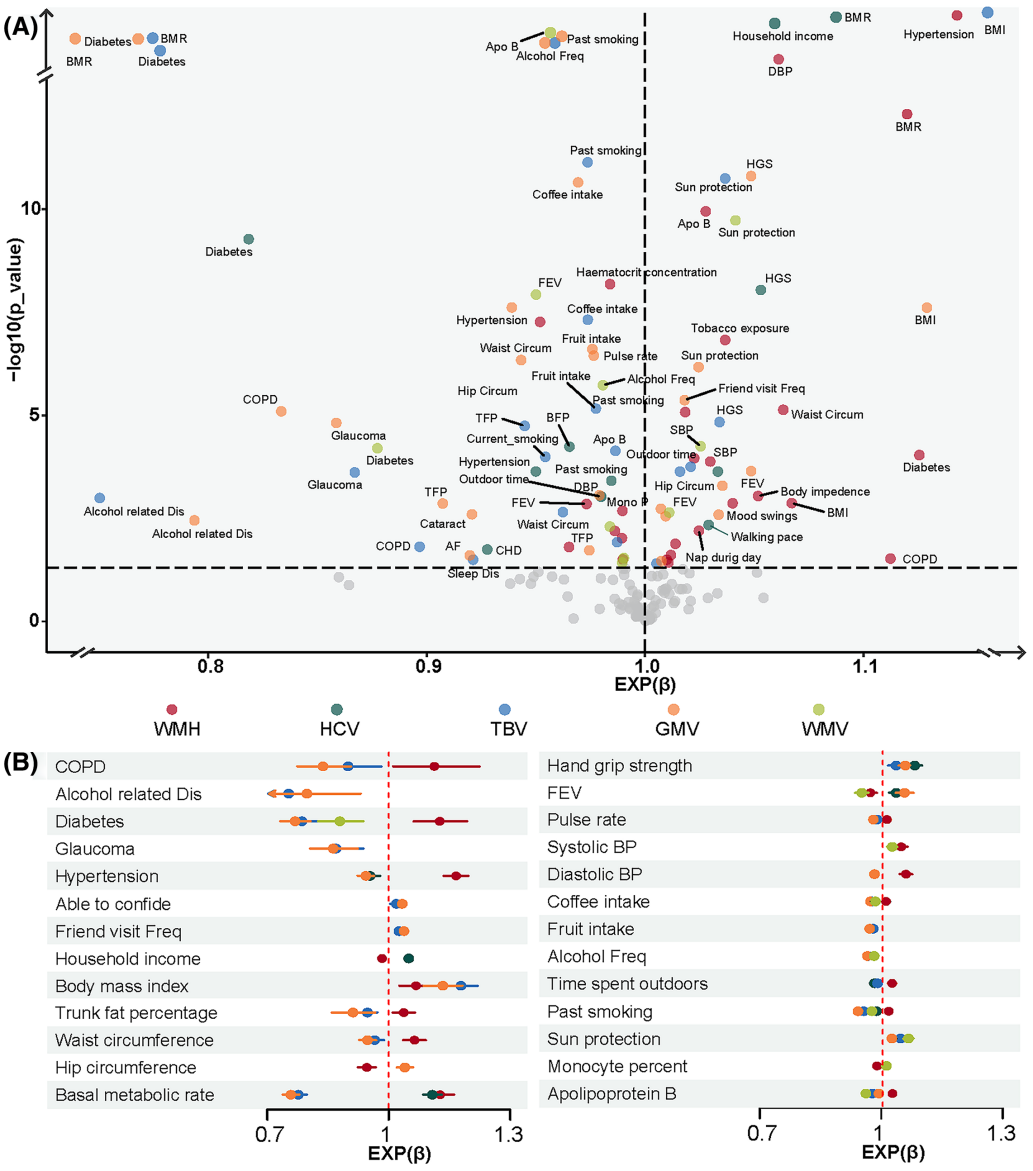
Associations between exposures and neuroimaging markers of brain health by LASSO regression analysis. (A). Factors associated with each neuroimaging marker of brain health. (B) Factors associated with at least two of the five neuroimaging markers of brain health. Abbreviations: AF, atrial fibrillation; BFP, body fat percentage; BMI, body mass index; BMR, basal metabolic rate; BP, blood pressure; CHD, chronic heart disease; COPD, chronic obstructive pulmonary disease; FEV, forced expiratory volume; GMV, gray matter volume; HCV, hippocampus volume; HGS, hand grip strength; TBV, total brain volume; TFP, trunk fat percentage; WMH, white matter hyperintensities; WMV, white matter volume.

As shown in Figure 2A, out of all the included exposures, 27, 11, 21, 26, and 10 factors were significantly associated with WMH, HCV, TBV, GMV, and WMV, respectively. Figure 2B highlights the validated factors associated with at least two of the five neuroimaging markers. Overall, systematic diseases (e.g., diabetes and hypertension) and unhealthy lifestyles (e.g., past/current smoking and heavy coffee intake) were significantly associated with larger WMH load and smaller brain volume. In contrast, well social supports (such as household income) were significantly associated with smaller WMH load and larger brain volume. In addition, measures of cardiopulmonary function (e.g., blood pressure and forced expiratory volume) and body size (such as fat percentage and waist circumference) were also associated with WMH load and brain volume. Regarding biochemical markers, a higher apolipoprotein B level was significantly associated with larger WMH load and smaller brain volume; inversely, a higher monocyte percentage was related to smaller WMH load and larger brain volume. More details are shown in Figure S2 and Table S5.

### 3.2 Restricted cubic splines

**FIGURE 3:**
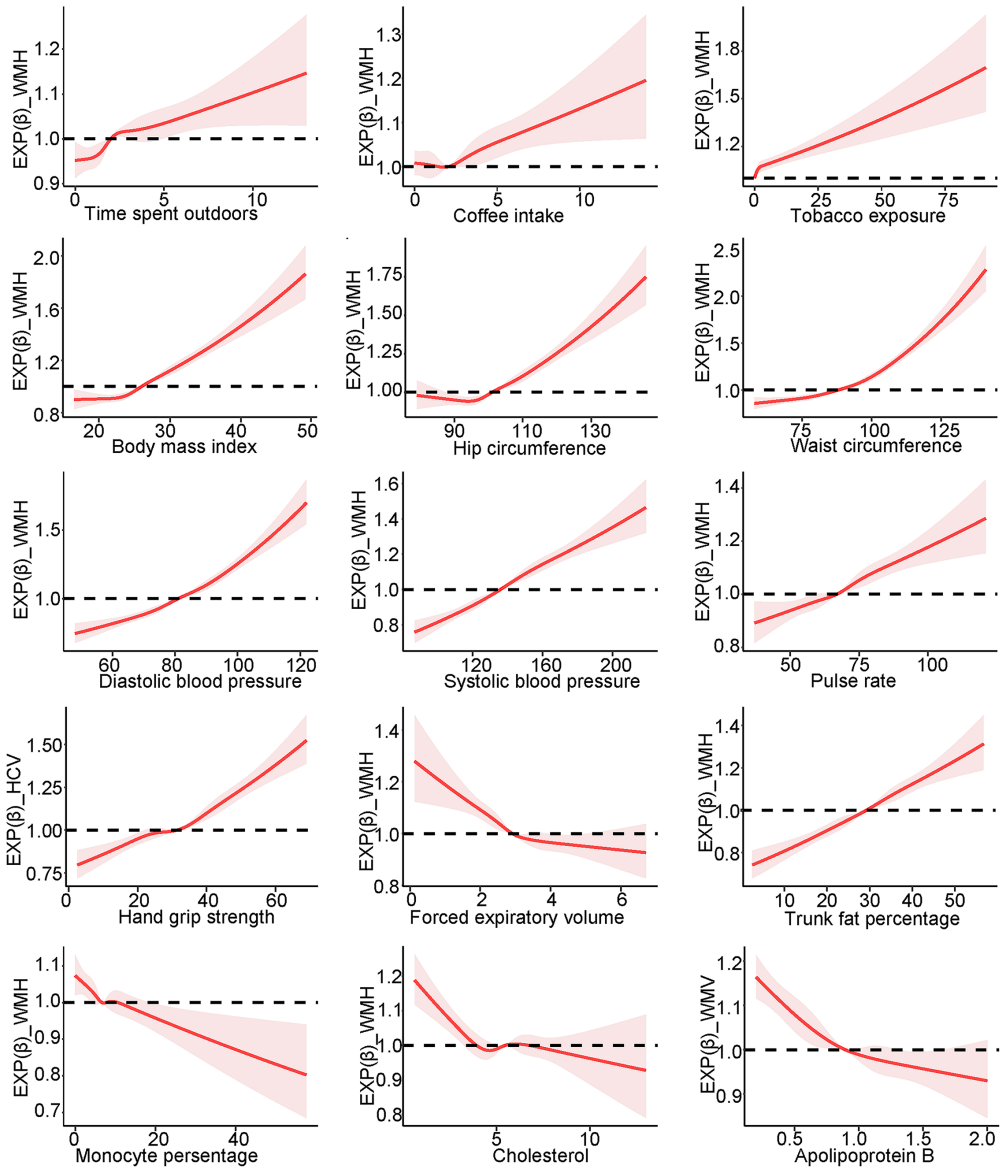
Nonlinear relationships between anthropometric indicators and imaging markers of brain health by restricted cubic splines. Horizontal axis indicates exposures; vertical axis indicates the exp‐transformed estimation of the correlations between exposures and imaging markers of brain health; the dotted line indicates the significance line (*p* = 0.05). Abbreviations: HCV, hippocampus volume; WMH, white matter hyperintensities; WMV, white matter volume.

Figure 3 depicts the nonlinear relationships between identified continuous variables and neuroimaging markers of brain health. Significant increases in WMH load were observed at unfavorable levels of anthropometric indicators, lifestyle factors, and biochemistry measures. Similarly, decreases in brain volume were also found at unfavorable levels of these exposures. The RCS analyses further identified a series of optimal ranges for these influencing factors to maintain brain health. For instance, avoiding tobacco exposure, spending no more than 2 h outdoors, and consuming no more than two cups of coffee per day were suggested to be beneficial to brain health. Additionally, maintaining moderate blood pressure and body size was also recommended to maintain brain health. More details are shown in Figure S3.

### 3.3 Weighted standardized scores and neuroimaging markers

A higher weighted standardized score was associated with smaller HCV, TBV, GMV, and WMV and a higher WMH load. The coefficient for HCV, TBV, GMV, WMV, and WMH associated with each standard deviation increment of the weighted standardized score was −3.30 (−3.43, −3.16), −1.25 (−1.35, −1.16), −1.43 (−1.51, −1.35), −1.81 (−2.10, −1.52), and 1.59 (1.52, 1.66), respectively. The percentage of the variance in HCV, TBV, GMV, WMV, and WMH load explained by multimorbidity score was 6.57%, 2.25%, 3.86%, 0.48%, and 6.04%, respectively (Table 1).

**TABLE 1:** Associations between weighted standardized scores and brain volumes.

	Quintile 1	Quintile 2	Quintile 3	*p* for trend	*R* ^2^
WMH					
*β* (95% CI), Model 1	Reference	0.025 (−0.002 to 0.052)	0.541 (0.514 to 0.568)	<0.001	0.061
*β* (95% CI), Model 2	Reference	0.145 (0.121 to 0.169)	0.298 (0.274 to 0.322)	<0.001	0.286
HCV					
*β* (95% CI), Model 1	Reference	−0.350 (−0.377 to −0.323)	−0.626 (−0.652 to −0.599)	<0.001	0.066
*β* (95% CI), Model 2	Reference	−0.116 (−0.144 to 0.089)	−0.203 (−0.234 to −0.172)	<0.001	0.155
TBV					
*β* (95% CI), Model 1	Reference	−0.226 (−0.253 to −0.199)	−0.301 (−0.328 to −0.274)	<0.001	0.023
*β* (95% CI), Model 2	Reference	−0.106 (−0.128 to −0.084)	−0.168 (−0.190 to −0.146)	<0.001	0.364
GMV					
*β* (95% CI), Model 1	Reference	−0.184 (−0.211 to −0.157)	−0.399 (−0.426 to −0.372)	<0.001	0.039
*β* (95% CI), Model 2	Reference	−0.112 (−0.132 to −0.091)	−0.242 (−0.262 to −0.221)	<0.001	0.469
WMV					
*β* (95% CI), Model 1	Reference	−0.042 (−0.070 to −0.014)	−2.950 (−2.956 to −2.944)	<0.001	0.005
*β* (95% CI), Model 2	Reference	−0.031 (−0.057 to −0.005)	−0.117 (−0.143 to −0.091)	<0.001	0.120

*Note*: Model 1 was the unadjusted model; Model 2 was adjusted for age and gender; WMH was log‐transformed in the analysis given its skewed distribution.

Abbreviations: CI, confidence interval; GMV, gray matter volume; HCV, hippocampus volume; TBV, total brain volume; WMH, white matter hyperintensity; WMV, white matter volume.

### 3.4 Subgroup analyses

**FIGURE 4:**
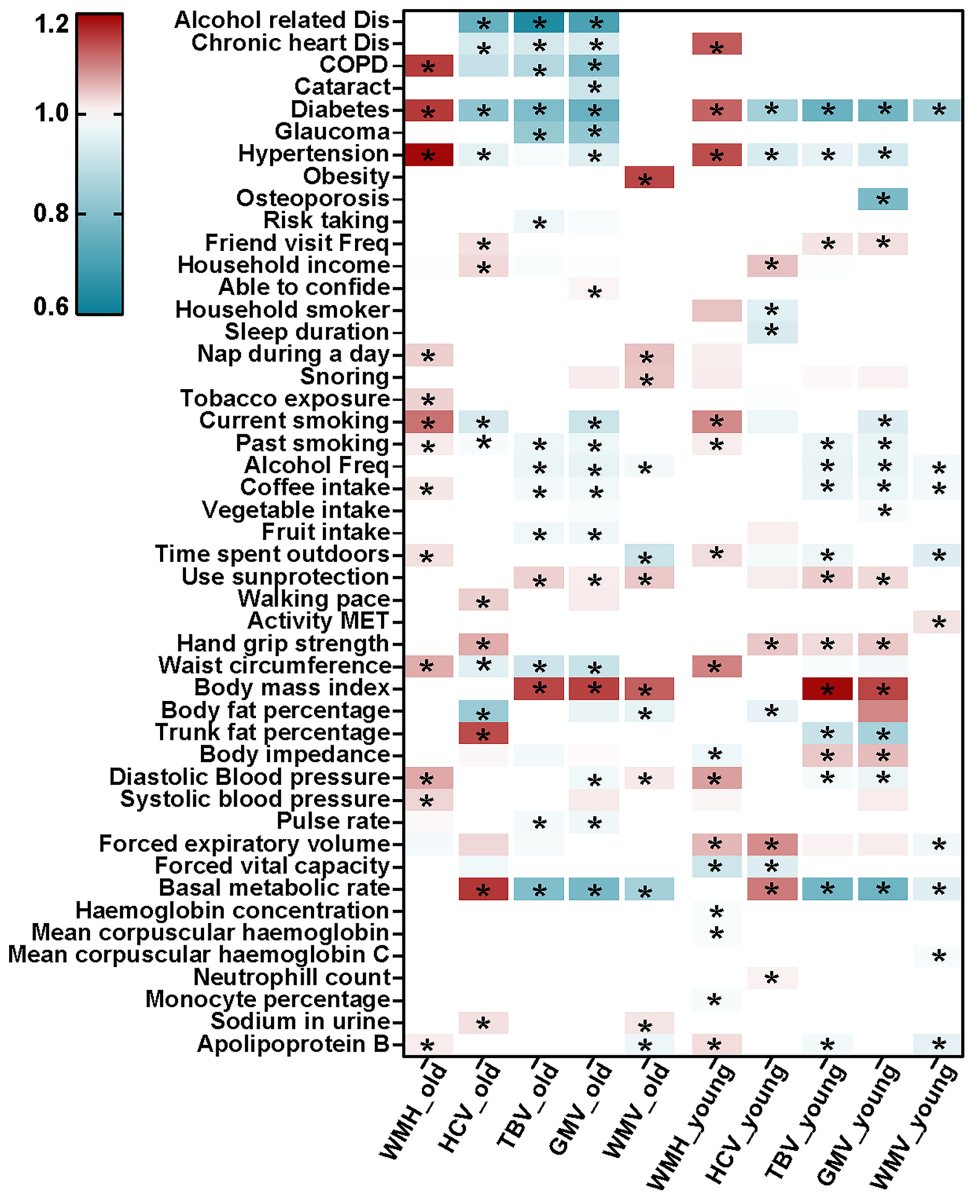
Subgroup analysis stratified by age. Abbreviations: COPD, chronic obstructive pulmonary disease; GMV, gray matter volume; HCV, hippocampus volume; TBV, total brain volume; WMH, white matter hyperintensities; WMV, white matter volume. *adjusted *p* < 0.05.

Age‐stratified subgroup analyses were also performed to explore potential heterogeneity. Although most of the associations remained significant among subgroups, we observed disparate results across ages. For example, the associations between systematic diseases and neuroimaging markers were stronger in older individuals compared to younger ones. Besides, a higher trunk fat percentage was associated with larger HCV in older individuals, while it was related to smaller TBV and GMV in younger ones. Further details are provided in Figure 4.

## 4 DISCUSSION

Through an exposome‐based approach, we identified exposures significantly linked to neuroimaging markers of brain health across six exposome categories: systematic diseases, lifestyle factors, social support, anthropometric measures, personality traits, and biochemical markers. Moreover, the percentage of the variance in HCV, TBV, GMV, WMV, and WMH load explained by multimorbidity score was 6.57%, 2.25%, 3.86%, 0.48%, and 6.04%, respectively.

Systematic diseases mostly reflecting cardiopulmonary dysfunction and metabolic disturbance (i.e., hypertension, diabetes) were significantly associated with smaller brain volume, as well as higher WMH load. Additionally, visual disturbances (cataracts and glaucoma) were also related to smaller brain volumes. These findings align with previous studies showing that cardiovascular diseases influenced cognitive aging trajectories in older adults^16^ that visual impairment increased the dementia risk while interventions to preserve vision reversed the risk^17^, and that sporadic AD derived mostly from various metabolic diseases such as diabetes.^18^, ^19^ Given the brain's vulnerability to impaired cerebral perfusion, the potential “head‐to‐heart & lung” link was largely attributed to diminished blood flow to the brain.^20^ Cerebral hypoperfusion was supposed to be an active contributor to the formation of both neurofibrillary tangles and amyloid plaques and an initiator of brain atrophy by causing acidosis and oxidative stress.^21^ Likewise, (neuro)inflammation played a crucial role in linking metabolic impairments to brain health, as elevated reactive oxygen species and nitric oxide production can lead to tissue damage and pathological protein aggregation.^22^ Consistent with previous studies,^10^, ^17^ a significant association between cataract and brain health was reported in the current research. A previous study reported that visual deprivation caused by visual impairments (such as cataracts) might induce retinal degeneration by decreasing levels of neurotrophic factors.^23^ The close link between visual impairment and degeneration of retinal and central visual pathway structures suggests that pathology in any of these components is likely to propagate along the entire chain through trans‐neuronal degeneration.^24^ Consequently, degeneration of visual pathway structures would ultimately result in cerebral atrophy and cognitive decline.

As our current results suggested, a wide range of poor lifestyle habits (e.g., high alcohol frequency, and smoking) were positively associated with smaller brain volume and higher WMH load. These findings align with previous research demonstrating that lifestyle factors play a significant role in the process of brain aging.^25^ On the one hand, alcohol, and nicotine could cause reduced cell density by inducing cell necrosis or apoptosis.^26^, ^27^ Additionally, caffeine‐induced morphological change in pyramidal cells and a reduction in information transmission can also cause brain volume changes.^28^ On the other hand, unhealthy lifestyles could indirectly impair brain health through systematic diseases. For example, the heavy consumption of unfiltered coffee, cigarettes, and alcohol can lead to lipid abnormalities,^29^, ^30^ a leading risk factor for atherosclerosis that was associated with AD.^31^ Previous studies reported that environmental sunlight could influence the physiological and cognitive functions by regulating the circadian rhythm and hormone secretion.^32^ Excessive absorption of ultraviolet radiation stimulated the production of reactive oxygen and nitrogen species, which could damage biological molecules (i.e., membrane lipids and deoxyribonucleic acid).^33^ Interestingly, another study reported a J‐shaped relationship between sunlight exposure and the risk of dementia.^34^ Thus, the association between sunlight and brain health might be mediated by sunlight intensity and sunlight exposure time.

Both higher trunk fat percentage and larger waist circumference were associated with smaller brain volume and higher WMH load in this study. However, the correlation between hip circumference and neuroimaging markers was opposite to that of waist circumference. This suggests that the actual effects of weight on brain health were determined by the location of fat accumulation because visceral fat and central obesity are linked to brain health by inducing cardiovascular disease.^35^, ^36^ High blood pressure was also associated with smaller brain volume and higher WMH load. Beyond cerebral atherosclerosis, oxidative damage, and reduced cerebral blood flow reported in previous research, hypoxia, and metabolic dysfunction have emerged as the emerging potential mechanisms underlying the association of unfavorable blood pressure and pulmonary function with brain health.^37^ Interestingly, the current study showed that higher trunk fat percentage was associated with larger brain volume in older while it was related to smaller brain volume in younger. Thus, the effect of fat percentage on brain health might be modified by age.

In the current study, mood swings were positively associated with WMH load. Personality traits, particularly emotional instability, are considered as a higher tendency to experience more stress in a variety of situations,^38^ which leads to chronic activation of the hypothalamic‐pituitary‐adrenal axis—a major contributory factor for brain disorders.^39^ It is well‐documented that lifestyle behaviors and personality traits are closely linked, and both are associated with brain integrity.^40^ Therefore, we hypothesized that the associations between personality traits and brain health could be mediated by lifestyle behaviors such as smoking, alcohol consumption, physical activity, and dietary habits. Moreover, the adverse effects of emotional instability might be attenuated by social support, as we found positive associations between the ability to confide in others and the frequency of friend visits with TBV and GMV.

Serum cholesterol pathway biomarkers (CPBs), including total cholesterol (TC), low‐density lipoprotein cholesterol (LDL‐C), high‐density lipoprotein cholesterol (HDL‐C), and apolipoproteins (e.g., apolipoprotein A‐I, apolipoprotein B), attracted widespread attention. A previous study reported that elevated LDL‐C (or apolipoprotein B) levels were associated with increased new T2 lesions.^41^ Consistent with this finding, we observed the detrimental role of apolipoprotein B in WMH load and brain atrophy. The associations between high apolipoprotein B levels and poor brain health might be mediated by inflammatory effects, as LDL‐C and apolipoprotein B can facilitate the production of pro‐inflammatory cytokines and tumor necrosis factor by immune cells.^42^ We also found significant associations between a higher monocyte percentage with a smaller WMH load and a larger WMV in this study. As a highly plastic immune cell, the monocyte has the ability to infiltrate the CNS and could switch its status between inflammatory and tissue remodeling.^43^ Though the role of monocytes cannot be simply described as beneficial or detrimental, monocytes are a promising target for immunotherapy or immunoregulation.

The findings of this study reveal a series of factors related to neuroimaging markers of brain health, which have broad implications for clinical practice. First, identifying modifiable lifestyle and health factors, such as hypertension, diabetes, smoking, and coffee consumption, can guide healthcare professionals in implementing targeted interventions to improve brain health. These interventions can be applied in everyday medical practice, especially in elderly patient populations. Second, this study emphasizes the importance of social support and mental health. Enhancing social support (such as increasing social activities) and improving mental well‐being can reduce the risk of brain damage. Therefore, healthcare providers can help maintain brain health by promoting social engagement and providing psychological support. Lastly, by monitoring biochemical markers (such as apolipoprotein B levels), healthcare practitioners can identify potential risks to brain health early and take preventive measures. These results highlight the need for a multidisciplinary approach, including collaboration across cardiovascular, endocrine, and mental health fields, to achieve comprehensive brain health management. In summary, the evidence provided by this study supports the application of preventive strategies in everyday clinical practice, focusing on modifying risk factors and enhancing social and psychological support to prevent brain structural damage and improve brain health.

Our research sought to reduce biases, including selective reporting, but several limitations must be considered when interpreting our findings. First, due to the cross‐sectional design, we were unable to establish causal relationships. Second, our EWAS was constrained by the available variables from the UKB database. Variables such as local environment were excluded due to the excessive missing values. Third, the weighted scores of the identified factors explain only a relatively small proportion of the variance in brain volumes, reflecting the complexity of brain structure determinants and highlighting the need to consider multiple factors in future studies to capture a greater share of the variance. Fourth, our analysis was limited to participants of the UK Biobank with MRI data. These participants tended to be younger, highly educated, and more likely to have healthier lifestyles compared to those without MRI data.^10^ As a result, our findings may not be generalizable to the entire UK population or other racial groups without further validation.

## 5 CONCLUSION

In conclusion, we summarized the variables associated with the neuroimaging markers of brain health across six primary exposome categories: systematic diseases, lifestyles, social supports, anthropometric measures, personality traits, and biochemical markers. These results highlight the potential of integrating diverse of exposures to devise effective strategies for preserving brain health.

## AUTHOR CONTRIBUTIONS

All authors had full access to the data in the study and accepted the responsibility to submit it for publication. J.‐T.Y. designed the study. L.‐Y.H. and Y.F. conducted the main analyses and drafted the manuscript. Y.Z., H.‐Y.H., L.‐Z.M., Y.‐J.G., Y.‐L.Z., Y.‐R.Z., and S.‐D.C. contributed to imaging and genetic data analyses. J.‐F.F., W.C., L.T., and J.‐T.Y. critically revised the manuscript, and all authors approved the final version.

## FUNDING INFORMATION

This study was supported by grants from the National Key R&D Program of China (2021YFC2500100), the Science and Technology Innovation 2030 Major Projects (2022ZD0211600), National Natural Science Foundation of China (82071201, 82071997), Shanghai Municipal Science and Technology Major Project (2018SHZDZX01), Research Start‐up Fund of Huashan Hospital (2022QD002), Excellence 2025 Talent Cultivation Program at Fudan University (3030277001), Shanghai Talent Development Funding for The Project (2019074), Shanghai Rising‐Star Program (21QA1408700), 111 Project (B18015), and ZHANGJIANG LAB, Tianqiao and Chrissy Chen Institute, the State Key Laboratory of Neurobiology and Frontiers Center for Brain Science of Ministry of Education, and Shanghai Center for Brain Science and Brain‐Inspired Technology, Fudan University.

## CONFLICT OF INTEREST STATEMENT

We declare no competing interests.

## Supporting information


Data S1.

## Data Availability

The main data used in this study were accessed from the publicly available UK Biobank Resource under application number 19542, which cannot be shared with other investigators. Any other data generated in the analysis process can be requested from the corresponding author.
