# Supplementary material for: Identifying modifiable factors associated with neuroimaging markers of brain health
Source: CNS Neurosci Ther. 2024 Oct 15;30(10):e70057. doi: 10.1111/cns.70057 (PMC11474882; doi:10.1111/cns.70057)

**Identifying Modifiable Factors Associated with Neuroimaging Markers of Brain Health**


| TITLE | PAGE |
|---|---|
| **Table S1. Modifiable factors included in this study.** | **2-7** |
| **Table S2. Ordinal variables included in this study.** | **8-9** |
| **Table S3. Continuous variables included in this study.** | **10-12** |
| **Table S4. The baseline demographic characteristics of participants.** | **13** |
| **Table S5. The significant factors identified by LASSO regression analysis.** | **14-15** |
| **Table S6. STROBE checklist of recommended items to address in cohort studies** | **16-18** |
| **Figure S1. The flowchart.** | **19** |
| **Figure S2. The significant factors identified by linear regression analysis.** | **20** |
| **Figure S3. The results of restrict cubic spline analyses.** | **21-24** |

**Table S1. Modifiable factors included in this study.**

| Exposure factors | Conditions included as reported by participants | Field ID |
|---|---|---|
| Atrial fibrillation | Atrial fibrillation and flutter | 131350 |
| Alcohol disorders | Mental and behavioral disorders due to use of alcohol | 130854 |
| Anemia | Iron deficiency anemia<br>Vitamin B12 deficiency anemia<br>Folate deficiency anemia<br>Other nutritional anemias<br>Anemia due to enzyme disorders<br>Thalassemia<br>Sickle-cell disorders<br>Other hereditary hemolytic anemias<br>Acquired hemolytic anemia<br>Acquired pure red cell aplasia [erythroblastosis]<br>Other aplastic anemias<br>Acute posthemorrhagic anemia<br>Anemia in chronic diseases classified elsewhere<br>Other anemias | 130622<br>130624<br>130626<br>130628<br>130630<br>130632<br>130634<br>130636<br>130638<br>130640<br><br>130642<br>130644<br><br>130646<br>130648 |
| Arrhythmias | other cardiac arrhythmias | 131352 |
| Arthritis | pyogenic arthritis<br>seropositive rheumatoid arthritis<br>other rheumatoid arthritis<br>juvenile arthritis<br>juvenile arthritis in diseases classified elsewhere<br>other arthritis | 131840<br>131848<br>131850<br>131854<br>131856<br><br>131864 |
| Asthma | asthma | 131494 |
| Atherosclerosis, aneurysm and peripheral vascular diseases | Atherosclerosis<br>Aortic aneurysm and dissection<br>Other aneurysm<br>Other peripheral vascular diseases<br>Arterial embolism and thrombosis | 131380<br>131382<br>131384<br>131386<br>131388 |
| Bronchiectasis | Bronchiectasis | 131498 |
| Cataract | Senile cataract<br>Other cataract<br>Cataract and other disorders of lens in diseases classified elsewhere | 131164<br>131166<br>131170 |
| Coronary heart disease | Angina pectoris<br>Acute myocardial infarction<br>Subsequent myocardial infarction<br>Certain current complications following acute myocardial infarction | 131296<br>131298<br>131300<br>131302 |

| | | |
|---|---|---|
| | Other acute ischemic heart diseases<br>Chronic ischemic heart disease | 131304<br>131306 |
| Chronic kidney disease | Chronic nephritic syndrome<br>Chronic tubulo-interstitial nephritis<br>Drug- and heavy-metal-induced tubulo-interstitial and tubular conditions<br>Chronic renal failure | 132004<br>132018<br>132024<br>132032 |
| Chronic Liver disease | Chronic viral hepatitis<br>Alcoholic liver disease<br>Toxic liver disease<br>Hepatic failure, not elsewhere classified<br>Chronic hepatitis, not elsewhere classified<br>Fibrosis and cirrhosis of liver<br>Other inflammatory liver diseases<br>Other diseases of liver | 130210<br>131658<br>131660<br>131662<br>131664<br>131666<br>131668<br>131670 |
| Chronic obstructive pulmonary disease | Simple and mucopurulent chronic bronchitis<br>Unspecified chronic bronchitis<br>Emphysema<br>Other chronic obstructive pulmonary disease | 131486<br>131488<br>131490<br>131492 |
| Dental caries | Dental caries | 131556 |
| Diabetes | Insulin-dependent diabetes mellitus<br>Non-insulin-dependent diabetes mellitus<br>Malnutrition-related diabetes mellitus<br>Other specified diabetes mellitus<br>Unspecified diabetes mellitus | 130706<br>130708<br>130710<br>130712<br>130714 |
| Dyspepsia | Dyspepsia | 131600 |
| Eating disorders | Eating disorders | 130918 |
| Gastritis , duodenitis and Oesophagitis | Oesophagitis<br>Gastritis and duodenitis | 131582<br>131598 |
| Glaucoma | Glaucoma | 131186 |
| Hearing loss | conductive and sensorineural hearing loss<br>other hearing loss | 131258<br>131260 |
| Heart failure | Cardiomyopathy<br>Heart failure | 131338<br>131354 |
| Hypertension | Essential (primary) hypertension<br>Hypertensive heart disease<br>Hypertensive renal disease<br>Hypertensive heart and renal disease<br>Secondary hypertension | 131286<br>131288<br>131290<br>131292<br>131294 |
| Hyperthyroidism | Thyrotoxicosis [hyperthyroidism] | 130700 |
| Hypothyroidism | Other hypothyroidism | 130696 |
| Inflammatory bowel disease | Crohn's disease [regional enteritis]<br>Ulcerative colitis | 131626<br>131628 |
| Irritable bowel | Irritable bowel syndrome | 131638 |

| | | |
|---|---|---|
| syndrome | | |
| Lipidemia | Disorders of lipoprotein metabolism and other lipidemia | 130814 |
| Obesity | Obesity | 130792 |
| Osteoporosis | Osteoporosis with pathological fracture<br>Osteoporosis without pathological fracture<br>Osteoporosis in diseases classified elsewhere | 131962<br>131964<br>131966 |
| Visual disturbances | visual disturbances<br>blindness and low vision | 131210<br>131212 |
| Sleep disorders | Sleep disorders<br>Nonorganic sleep disorders | 131060<br>130920 |
| Tobacco disorders | Mental and behavioral disorders due to use of tobacco | 130868 |
| Ulcer | Gastric ulcer<br>Duodenal ulcer<br>Peptic ulcer, site unspecified<br>Gastrojejunal ulcer | 131590<br>131592<br>131594<br>131596 |
| Visual disturbances | visual disturbances<br>blindness and low vision | 131210<br>131212 |
| Valve disorders | rheumatic mitral valve diseases<br>rheumatic aortic valve diseases<br>rheumatic tricuspid valve diseases<br>multiple valve diseases<br>nonrheumatic mitral valve disorders<br>nonrheumatic aortic valve disorders<br>nonrheumatic tricuspid valve disorders<br>pulmonary valve disorders<br>endocarditis, valve unspecified<br>endocarditis and heart valve disorders in diseases classified elsewhere | 131276<br>131278<br>131280<br>131282<br>131322<br>131324<br>131326<br>131328<br>131330<br>131332 |
| **Lifestyle grouping** | | |
| Smoking | Current tobacco smoking<br>Past tobacco smoking<br>Smoking/smokers in household<br>Exposure to tobacco smoke at home<br>Exposure to tobacco smoke outside home<br>Ever smoked | 1239<br>1249<br>1259<br>1269<br>1279<br>20160 |
| Alcohol | Alcohol drinking status<br>Alcohol intake frequency | 20117<br>1558 |
| Activity | Duration of moderate activity<br>MET minutes per week for moderate activity<br>MET minutes per week for vigorous activity<br>Summed MET minutes per week for all activity<br>Summed days activity<br>Summed minutes activity | 894<br>22038<br>22039<br>22040<br>22033<br>22034 |

| | | |
|---|---|---|
| | IPAQ activity group | 22032 |
| Food | Cooked vegetable intake<br>Salad/raw vegetable intake<br>Fresh fruit intake<br>Dried fruit intake<br>Oily fish intake<br>Non-oily fish intake<br>Tea intake<br>Coffee intake<br>Variation in diet<br>Processed meat intake<br>Poultry intake<br>Beef intake<br>Lamb intake<br>Pork intake | 1289<br>1299<br>1309<br>1319<br>1329<br>1339<br>1488<br>1498<br>1548<br>1349<br>1359<br>1369<br>1379<br>1389 |
| Sleep | Sleep duration<br>Nap during a day<br>Snoring | 1160<br>1190<br>1210 |
| Behaviors | Use of sun protection<br>Usual walking pace<br>Time spend outdoors in summer<br>Time spend outdoors in winter | 2267<br>924<br>1050<br>1060 |
| **Social support and so on** | | |
| Social support | Frequency of friend visits<br>Able to confide<br>Average total household income | 1031<br>2110<br>738 |
| **Anthropometric indicators** | | |
| Body size and body shape | Body mass index<br>Weight<br>Waist circumference<br>Hip circumference<br>Hand grip strength (left)<br>Hand grip strength (right)<br>Trunk fat percentage<br>Basal metabolic rate<br>Body fat percentage<br>Impedance of whole body | 21001<br>21002<br>48<br>49<br>46<br>47<br>23127<br>23105<br>23099<br>23106 |
| Blood pressure | Diastolic blood pressure<br>Systolic blood pressure<br>Pulse rate | 4079<br>4080<br>102 |
| Pulmonary function test | Forced vital capacity (FVC)<br>Forced expiratory volume in 1-second (FEV1)<br>Peak expiratory flow (PEF)<br>FEV1/ FVC ratio Z-score | 3062<br>3063<br>3064<br>20258 |

| | | |
|---|---|---|
| **Personality traits** | | |
| Feelings and frequency of feelings | Mood swings | 1920 |
| | Miserableness | 1930 |
| | Irritability | 1940 |
| | Sensitivity/hurt feelings | 1950 |
| | Fed-up feeling | 1960 |
| | Nervous feelings | 1970 |
| | Worry/anxious feelings | 1980 |
| | Tense/highly strung | 1990 |
| | Worry too long after embarrassment | 2000 |
| | Suffer from nerves | 2010 |
| | Loneliness isolation | 2020 |
| | Guilty feelings | 2030 |
| | Risk taking | 2040 |
| | Frequency of depressed mood | 2050 |
| | Frequency of unenthusiasm | 2060 |
| | Frequency of tenseness / restlessness | 2070 |
| | Frequency of tiredness / lethargy | 2080 |
| **blood sample examinations** | | |
| Blood count | White blood cell | 30000 |
| | Red blood cell | 30010 |
| | Hemoglobin concentration | 30020 |
| | Hemoglobin percentage | 30030 |
| | Platelet count | 30080 |
| | Lymphocyte count | 30120 |
| | Monocyte count | 30130 |
| | Neutrophil count | 30140 |
| | Eosinophil count | 30150 |
| | Basophil count | 30160 |
| | Lymphocyte percentage | 30180 |
| | Monocyte percentage | 30190 |
| | Neutrophil percentage | 30200 |
| | Eosinophil percentage | 30210 |
| | Basophil percentage | 30220 |
| | Reticulocyte count | 30150 |
| blood biochemistry | Albumin | 30600 |
| | Alkaline phosphatase | 30610 |
| | Alanine aminotransferase (ALT) | 30620 |
| | Apolipoprotein A | 30630 |
| | Apolipoprotein B | 30640 |
| | Aspartate aminotransferase (AST) | 30650 |
| | Direct bilirubin | 30660 |
| | Urea | 30670 |
| | Calcium | 30680 |

| | | |
|---|---|---|
| | Cholesterol | 30690 |
| | Creatinine | 30700 |
| | C-reactive protein | 30710 |
| | cystainC | 30720 |
| | Gamma glutamyltransferase | 30730 |
| | Glycated haemoglobin (HbA1c) | 30750 |
| | Insulin-Like Growth Factor | 30770 |
| | Low density lipoprotein | 30780 |
| | Total bilirubin | 30840 |
| | Triglyceride | 30870 |
| | Urate | 30880 |
| | Vitamin D | 30890 |

**Table S2. Ordinal variables included in this study.**

| Exposure | Data coding | Field ID |
|---|---|---|
| Past tobacco-smoking | 1 never<br>2 once or twice<br>3 occasionally<br>4 almost every day | 1249 |
| Current tobacco-smoking | 1 no<br>2 occasionally<br>3 almost every day | 1239 |
| Frequency of drinking alcohol | 1 never<br>2 occassionally<br>3 one or three time a month<br>4 one or twice a week<br>5 three of four time a week<br>6 daily | 1558 |
| IPAQ activity group | 1 low<br>2 moderate<br>3 high | 22032 |
| Variation in diet | 1 never<br>2 sometimes<br>3 often | 1548 |
| Nap during a day | 1 never/rarely<br>2 sometimes<br>3 usually | 1190 |
| Frequency of friend visits | 1 almost never<br>2 once every few month<br>3 once a month<br>4 once a week<br>5 2-4 times a week<br>6 almost every day | 1031 |
| Able to confide | 1 almost never<br>2 once every few month<br>3 once a month<br>4 once a week<br>5 2-4 times a week<br>6 almost every day | 2110 |
| Use of sun-protection | 1 never<br>2 sometimes<br>3 usually<br>4 always | 2267 |
| Average household income | 1 less than 18000<br>2 18000-30999<br>3 31000-51999<br>4 52000-100000 | 738 |

| | | |
|---|---|---|
| | 5 more than 100000 | |
| Sleep duration | 1.<7 or >=9 hours/day | 1160 |
| Usual walking pace | 1 slow<br>2 steady<br>3 brisk | 924 |

**Table S3. Continuous variables included in this study.**

| Exposure | Unit | Field ID |
|---|---|---|
| Hand grip strength (left) | Kg | 46 |
| Hand grip strength (right) | Kg | 47 |
| Hand grip strength | Kg | ID46 + ID47 |
| Waist circumference | cm | 48 |
| Hip circumference | cm | 49 |
| Diastolic blood pressure | mmHg | 4079 |
| Systolic blood pressure | mmHg | 4080 |
| Paulse rate | bpm | 102 |
| Forced vital capacity (FVC) | litres/min | 3062 |
| Forced expiratory volume in 1-second (FEV1) | litres/min | 3063 |
| Peak expiratory flow (PEF) | litres/min | 3064 |
| Tobacco exposure at home | hours/week | 1269 |
| Tobacco exposure outside home | hours/week | 1279 |
| Tobacco exposure | hours/week | ID1269 + ID1279 |
| Cooked vegetable intake | tablespoons/day | 1289 |
| Raw vegetable intake | tablespoons/day | 1299 |
| Vegetable intake | tablespoons/day | ID1289 + ID1299 |
| Fresh fruit intake | pieces/day | 1309 |
| Dried fruit intake | pieces/day | 1319 |
| Fruit intake | pieces/day | ID1309 + ID1319 |
| Processed meat intake | pieces/day | 1349 |
| Poultry intake | pieces/day | 1359 |
| Beef intake | pieces/day | 1369 |
| Lamb intake | pieces/day | 1379 |
| Pork intake | pieces/day | 1389 |
| Meat intake | pieces/day | ID1349+ID1359+ID1369+ ID1379+ID1389 |
| Tea intake | cups/day | 1488 |
| Coffee intake | cups/day | 1498 |
| Time spent outdoors in summer | hours/day | 1050 |
| Time spent outdoors in winter | hours/day | 1060 |
| Time spent outdoors | hours/day | ID1050 + ID1060 |
| Duration of moderate activity | minutes/day | 894 |
| Duration of vigorous activity | minutes/day | 914 |
| Duration of moderate to vigorous activity | minutes/day | ID894 + ID914 |
| Body mass index | Kg/m2 | 21001 |
| weight | Kg | 21002 |
| Trunk fat percentage | percent | 23127 |
| Basal metabolic rate | KJ | 23105 |
| Body fat percentage | percent | 23099 |

| | | |
|---|---|---|
| Body impedance | ohms | 23106 |
| Summed days activity | NA | 23033 |
| Summed minutes activity | NA | 23034 |
| MET minute per week for moderate activity | Minutes per week | 22038 |
| MET minute per week for vigorous activity | Minutes per week | 22039 |
| MET minute per week for moderate to vigorous activity | Minutes per week | ID22038 + ID22039 |
| Summed MET minutes for all activity | Minutes per week | 22040 |
| Deprivation index | NA | 26410 |
| Income score | NA | 26411 |
| Employment score | NA | 26412 |
| Health score | NA | 26413 |
| Education score | NA | 26414 |
| Housing score | NA | 26415 |
| Crime score | NA | 26416 |
| Living score | NA | 26417 |
| White blood cell | 10^9 cells/Litre | 30000 |
| Red blood cell | 10^9 cells/Litre | 30010 |
| Hemoglobin concentration | 10^9 cells/Litre | 30020 |
| Hemoglobin percentage | 10^9 cells/Litre | 30030 |
| Platelet count | 10^9 cells/Litre | 30080 |
| Lymphocyte count | 10^9 cells/Litre | 30120 |
| Monocyte count | 10^9 cells/Litre | 30130 |
| Neutrophil count | 10^9 cells/Litre | 30140 |
| Eosinophil count | 10^9 cells/Litre | 30150 |
| Basophil count | 10^9 cells/Litre | 30160 |
| Lymphocyte percentage | percent | 30180 |
| Monocyte percentage | percent | 30190 |
| Neutrophil percentage | percent | 30200 |
| Eosinophil percentage | percent | 30210 |
| Basophil percentage | percent | 30220 |
| Reticulocyte count | 10^12 cells/Litre | 30150 |
| Albumin | g/L | 30600 |
| Alkaline phosphatase | U/L | 30610 |
| Alanine aminotransferase (ALT) | U/L | 30620 |
| Apolipoprotein A | g/L | 30630 |
| Apolipoprotein B | g/L | 30640 |
| Aspartate aminotransferase (AST) | U/L | 30650 |
| Direct bilirubin | umol/L | 30660 |
| Urea | mmol/L | 30670 |
| Calcium | mmol/L | 30680 |
| Cholesterol | mmol/L | 30690 |
| Creatinine | umol/L | 30700 |

| | | |
|---|---|---|
| C-reactive protein | mg/L | 30710 |
| Cystatin C | mg/L | 30720 |
| Gamma glutamyltransferase | U/L | 30730 |
| Glycated haemoglobin (HbA1c) | mmol/mol | 30750 |
| Insulin-Like Growth Factor | nmol/L | 30770 |
| Low density lipoprotein | mmol/L | 30780 |
| Total bilirubin | umol/L | 30840 |
| Triglyceride | mmol/L | 30870 |
| Urate | umol/L | 30880 |
| Vitamin D | nmol/L | 30890 |

**Table S4. The baseline demographic characteristics of participants**

| **Characteristic** | **All participants (n=30,651)** | **Female (n=15,615)** | **Male (n=15,036)** | ***P*** |
|---|---|---|---|---|
| Age during MRI | 64.34 (7.69) | 63.66 (7.56) | 65.04 (7.77) | **<0.001** |
| Smoking, n (%) | 11,885 (38.78) | 5,510 (35.29) | 6,375 (42.40) | **<0.001** |
| Heavy drinking, n (%) | 6,966 (22.73) | 2,803 (17.95) | 4,163 (27.69) | **<0.001** |
| Hypertension, n (%) | 6,520 (21.27) | 2,613 (16.73) | 3,907 (25.98) | **<0.001** |
| Coronary heart disease, n (%) | 954 (3.11) | 246 (1.58) | 708 (4.71) | **<0.001** |
| Diabetes, n (%) | 893 (2.91) | 270 (1.73) | 623 (4.14) | **<0.001** |
| Body mass index | 26.58 (4.19) | 26.06 (4.53) | 27.12 (3.72) | **<0.001** |
| Systolic blood pressure, mmHg | 137.30 (18.77) | 134.10 (19.19) | 140.7 (17.69) | **<0.001** |
| Diastolic blood pressure, mmHg | 81.50 (10.39) | 79.65 (10.26) | 83.41 (10.16) | **<0.001** |
| White matter hyperintensity (log) | 8.05 (1.01) | 7.95 (0.99) | 8.15 (1.01) | **<0.001** |
| Total brain volume $*10^6$ | 1.49 (0.74) | 1.50 (0.74) | 1.48 (0.72) | **<0.001** |
| Hippocampal volume(left) $*10^3$ | 3.77 (0.49) | 3.68 (0.44) | 3.86 (0.52) | **<0.001** |
| Hippocampal volume(right) $*10^3$ | 3.88 (0.50) | 3.78 (0.44) | 3.99 (0.54) | **<0.001** |
| White matter volume $*10^5$ | 6.98 (4.12) | 6.97 (4.08) | 7.04 (4.12) | **<0.001** |
| Grey matter volume $*10^5$ | 8.05 (4.84) | 8.06 (4.61) | 7.74 (4.53) | **<0.001** |

Descriptive statistics were presented as mean (standard deviation) for continuous variables and number (percentage) for categorical variables. The t tests were used to compare means, and $\chi 2$ tests were used for binary variables in the Table.

**Table S5. The significant factors identified by LASSO regression analysis.**

| Exposures | EXP(β)_WMH | P_WMH | EXP(β)_HCV | P_HCV | EXP(β)_TBV | P_TBV | EXP(β)_GMV | P_GMV | EXP(β)_WMV | P_WMV |
|---|---|---|---|---|---|---|---|---|---|---|
| Atrial fibrillation | NA | NA | NA | NA | NA | NA | 0.92 | 2.49E-02 | NA | NA |
| CHD | NA | NA | 0.928 | 1.80E-02 | NA | NA | NA | NA | NA | NA |
| COPD | 1.112 | 2.99E-02 | NA | NA | 0.897 | 1.54E-02 | 0.833 | 8.02E-06 | NA | NA |
| Alcohol related Dis | NA | NA | NA | NA | 0.75 | 1.01E-03 | 0.794 | 3.54E-03 | NA | NA |
| Cataract | NA | NA | NA | NA | NA | NA | 0.921 | 2.52E-03 | NA | NA |
| Diabetes | 1.126 | 9.21E-05 | 0.818 | 5.32E-10 | 0.783 | 6.95E-19 | 0.764 | 1.27E-26 | 0.877 | 6.28E-05 |
| Glaucoma | NA | NA | NA | NA | 0.867 | 2.43E-04 | 0.859 | 1.53E-05 | NA | NA |
| Hypertension | 1.166 | 5.85E-30 | 0.95 | 2.31E-04 | NA | NA | 0.939 | 2.42E-08 | NA | NA |
| Sleep disorder | NA | NA | NA | NA | 0.921 | 3.18E-02 | NA | NA | NA | NA |
| Mood swings | 1.04 | 1.36E-03 | NA | NA | NA | NA | NA | NA | NA | NA |
| Able to confide | NA | NA | NA | NA | 1.005 | 3.96E-02 | 1.007 | 1.86E-03 | NA | NA |
| Friend visit Freq | NA | NA | NA | NA | 1.016 | 2.32E-04 | 1.018 | 4.27E-06 | NA | NA |
| Household income | 0.99 | 3.13E-02 | 1.046 | 7.37E-19 | NA | NA | NA | NA | 0.99 | 3.72E-02 |
| Body mass index | 1.067 | 1.35E-03 | NA | NA | 1.176 | 1.64E-19 | 1.129 | 2.46E-08 | NA | NA |
| Body fat percent | NA | NA | 0.965 | 5.76E-05 | NA | NA | NA | NA | NA | NA |
| Trunk fat percent | 0.965 | 1.57E-02 | NA | NA | 0.945 | 1.80E-05 | 0.907 | 1.38E-03 | NA | NA |
| Body impedance | 1.052 | 9.08E-04 | NA | NA | NA | NA | NA | NA | NA | NA |
| Waist circumference | 1.063 | 7.35E-06 | NA | NA | 0.962 | 2.23E-03 | 0.943 | 4.57E-07 | NA | NA |
| Hip circumference | 0.946 | 3.20E-06 | NA | NA | NA | NA | 1.035 | 5.10E-04 | NA | NA |
| BMR | 1.12 | 4.95E-13 | 1.111 | 2.71E-22 | 0.774 | 1.05E-73 | 0.754 | 1.22E-88 | NA | NA |
| Hand grip strength | NA | NA | 1.053 | 9.12E-09 | 1.034 | 1.46E-05 | 1.049 | 1.58E-11 | NA | NA |
| FEV | 0.973 | 1.41E-03 | 1.033 | 2.32E-04 | NA | NA | 1.034 | 2.57E-03 | 0.95 | 1.18E-08 |

| | | | | | | | | | | |
|---|---|---|---|---|---|---|---|---|---|---|
| FVC | NA | NA | NA | NA | NA | NA | 0.975 | 1.88E-02 | NA | NA |
| Pulse rate | 1.014 | 1.31E-02 | NA | NA | 0.987 | 1.20E-02 | 0.977 | 3.57E-07 | NA | NA |
| Systolic BP | 1.03 | 1.32E-04 | NA | NA | NA | NA | NA | NA | 1.026 | 5.64E-05 |
| Diastolic BP | 1.061 | 5.45E-15 | NA | NA | NA | NA | 0.979 | 8.69E-04 | NA | NA |
| Coffee intake | 1.012 | 2.46E-02 | NA | NA | 0.974 | 4.81E-08 | 0.969 | 2.24E-11 | 0.984 | 4.97E-03 |
| Fruit intake | NA | NA | NA | NA | 0.977 | 6.87E-06 | 0.976 | 2.48E-07 | NA | NA |
| Alcohol Freq | NA | NA | NA | NA | 0.964 | 9.42E-26 | 0.962 | 1.72E-34 | 0.981 | 1.86E-06 |
| Time spent outdoors | 1.023 | 1.10E-04 | 0.98 | 9.36E-04 | 1.021 | 1.77E-04 | NA | NA | NA | NA |
| Tobacco exposure | 1.037 | 1.49E-07 | NA | NA | NA | NA | NA | NA | NA | NA |
| Current smoking | NA | NA | NA | NA | 0.954 | 1.02E-04 | NA | NA | NA | NA |
| Past smoking | 1.018 | 8.36E-06 | 0.985 | 3.86E-04 | 0.974 | 7.28E-12 | 0.965 | 2.84E-25 | 0.99 | 2.89E-02 |
| Sun protection | NA | NA | NA | NA | 1.037 | 1.80E-11 | 1.025 | 6.76E-07 | 1.041 | 1.87E-10 |
| Nap during a day | 1.025 | 6.29E-03 | NA | NA | NA | NA | NA | NA | NA | NA |
| Walking pace | NA | NA | 1.029 | 4.55E-03 | NA | NA | NA | NA | NA | NA |
| MCHC | 0.984 | 6.60E-09 | NA | NA | NA | NA | NA | NA | NA | NA |
| Monocyte percent | 0.99 | 2.07E-03 | NA | NA | NA | NA | NA | NA | 1.011 | 2.29E-03 |
| Alkaline phosphatase | 1.011 | 3.71E-02 | NA | NA | NA | NA | NA | NA | NA | NA |
| Apolipoprotein B | 1.028 | 1.13E-10 | NA | NA | 0.986 | 7.26E-05 | 1.009 | 2.81E-03 | 0.963 | 2.15E-21 |
| Cholesterol | 0.986 | 6.39E-03 | NA | NA | NA | NA | NA | NA | NA | NA |
| C-reactive protein | 0.989 | 9.56E-03 | NA | NA | NA | NA | NA | NA | NA | NA |
| IGF-1 | NA | NA | NA | NA | NA | NA | 1.007 | 3.44E-02 | NA | NA |
| Total bilirubin | 1.01 | 3.18E-02 | NA | NA | NA | NA | NA | NA | NA | NA |

Abbreviations: WMH, white matter hyperintensities; TBV, total brain volume; HCV, hippocampus volume; WMV, white matter volume; GMV, grey matter volume; COPD, chronic obstructive pulmonary disease; BP, blood pressure; FEV, forced expiratory volume; FVC, forced vital capacity; BMR, basal metabolic rate; Mean corpuscular hemoglobin concentration; IGF, Insulin-like growth factor

**Table S6: STROBE checklist of recommended items to address in cohort studies.**

| Item No. | Section | Checklist item | Where addressed in manuscript |
|---|---|---|---|
| **1** | **TITLE and ABSTRACT** | (a) Indicate the study's design with a commonly used term in the title or the abstract | Title |
| | | (b) Provide in the abstract an informative and balanced summary of what was done and what was found | Abstract |
| | **INTRODUCTION** | | |
| **2** | **Background/rationale** | Explain the scientific background and rationale for the investigation being reported | Introduction, paragraph 1-2 |
| **3** | **Objectives** | State specific objectives, including any prespecified hypotheses | Introduction, paragraph 3 |
| | **METHODS** | | |
| **4** | **Study design and data sources** | Present key elements of study design early in the paper | Introduction (paragraph 3), Fig 1, Methods, paragraph 1 |
| **5** | **Setting** | Describe the setting, locations, and relevant dates, including periods of recruitment, exposure, follow-up, and data collection | Methods, paragraphs 1 |
| **6** | **Participants** | (a) Give the eligibility criteria, and the sources and methods of selection of participants. Describe methods of follow-up | Methods- Participants, paragraph 1 |
| | | (b) For matched studies, give matching criteria and number of exposed and unexposed | Not applicable |
| **7** | **Variables** | Clearly define all outcomes, exposures, predictors, potential confounders, and effect modifiers. Give diagnostic criteria, if applicable | Methods- Variables, paragraphs 2-3 |
| **8** | **Data sources/measurement** | For each variable of interest, give sources of data and details of methods of assessment (measurement). Describe comparability of assessment methods if there is more than one group | Methods- Variables, paragraphs 2-3 |
| **9** | **Bias** | Describe any efforts to address potential sources of bias | Methods- Variables, paragraphs 2-3 |
| **10** | **Study size** | Explain how the study size was arrived at | Methods- Participants, paragraph 1; Fig S1 |
| **11** | **Quantitative variables** | Explain how quantitative variables were handled in the analyses. If applicable, describe which groupings were | Methods- Variables, paragraphs 2-3 |

| | | | |
|---|---|---|---|
| | | chosen and why | |
| **12** | **Statistical methods** | (a) Describe all statistical methods, including those used to control for confounding | Methods-Statistical analyses, paragraph 1 |
| | | (b) Describe any methods used to examine subgroups and interactions | Methods-Statistical analyses, paragraph 2 |
| | | (c) Explain how missing data were addressed | Methods-Statistical analyses, paragraph 1 |
| | | (d) If applicable, explain how loss to follow-up was addressed | Not applicable |
| | | (e) Describe any sensitivity analyses | Methods-Statistical analyses, paragraph 2-3 |
| | **RESULTS** | | |
| **13** | **Participants** | (a) Report numbers of individuals at each stage of study—eg numbers potentially eligible, examined for eligibility, confirmed eligible, included in the study, completing follow-up, and analysed | Table S4 |
| | | (b) Give reasons for non-participation at each stage | Fig S1 |
| | | (c) Consider use of a flow diagram | Fig S1 |
| **14** | **Descriptive data** | (a) Give characteristics of study participants (eg demographic, clinical, social) and information on exposures and potential confounders | Results, paragraph 1, Table S4 |
| | | (b) Indicate number of participants with missing data for each variable of interest | Fig 1; Fig S1 |
| | | (c) Summarise follow-up time (eg, average and total amount) | Relevant for cognition online only (in abstract and methods, clinical measures) |
| **15** | **Outcome data** | Report numbers of outcome events or summary measures over time | Results, Fig 2 |
| | | c) If relevant, consider translating estimates of relative risk into absolute risk for a meaningful time period | Not applicable |
| | | d) Consider plots to visualize results (e.g. forest plot, scatterplot of associations between genetic variants and outcome versus between genetic variants and exposure) | Fig 2 & Fig 3 & Fig 4 |
| **15** | **Main results** | (*a*) Give unadjusted estimates and, if applicable, confounder-adjusted estimates and their precision (eg, 95% confidence interval). Make clear which confounders were adjusted for and why they were included | Results, paragraph 2-5 |

| | | | |
|---|---|---|---|
| | | (b) Report category boundaries when continuous variables were categorized | Results, paragraph 2-5 |
| | | (*c*) If relevant, consider translating estimates of relative risk into absolute risk for a meaningful time period | Results, paragraph 2-5 |
| | | b) Report results from other sensitivity analyses or additional analyses | Not applicable |
| **17** | **Other analyses** | Report other analyses done—eg analyses of subgroups and interactions, and sensitivity analyses | Fig S3 |
| | **DISCUSSION** | | |
| **18** | **Key results** | Summarise key results with reference to study objectives | Discussion, paragraph 1 |
| **19** | **Limitations** | Discuss limitations of the study, taking into account sources of potential bias or imprecision. Discuss both direction and magnitude of any potential bias | Discussion, paragraphs 8 |
| **20** | **Interpretation** | Give a cautious overall interpretation of results considering objectives, limitations, multiplicity of analyses, results from similar studies, and other relevant evidence | Discussion, paragraphs 2-6 |
| **21** | **Generalisability** | Discuss the generalisability (external validity) of the study results | Discussion, paragraphs 7-8 |
| | **OTHER INFORMATION** | | |
| **22** | **Funding** | Give the source of funding and the role of the funders for the present study and, if applicable, for the original study on which the present article is based | Financial disclosure |

**Figure S1. The flowchart.**

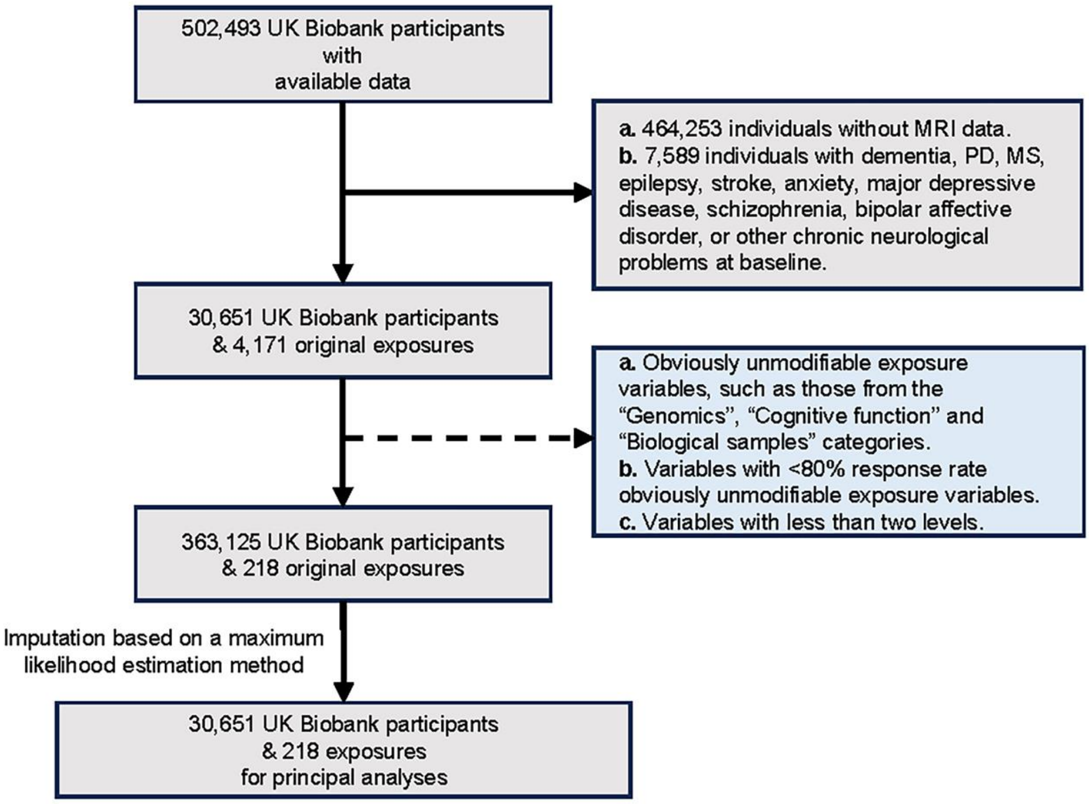

**Figure S2. The significant factors identified by linear regression analysis.**

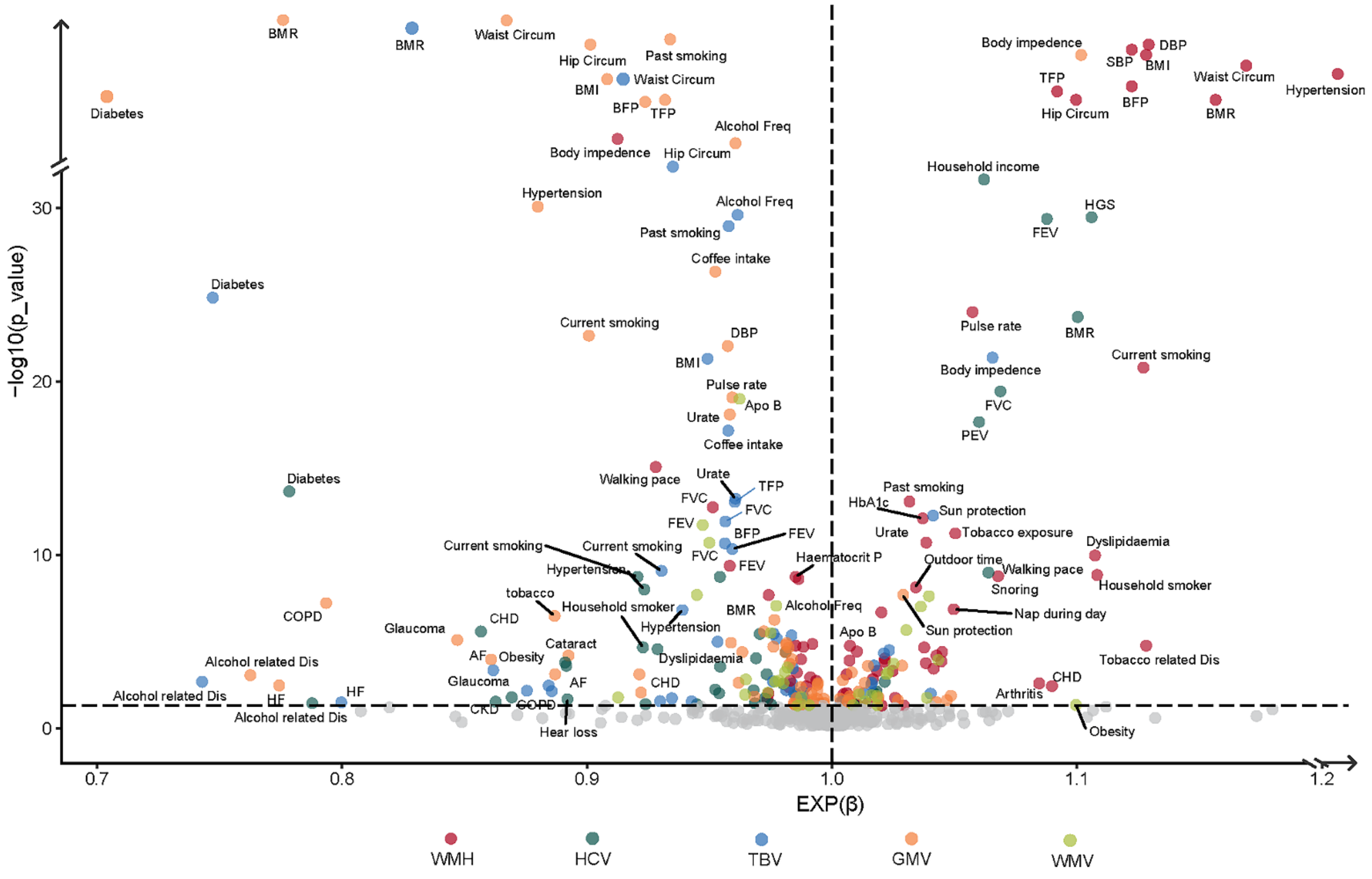


Abbreviation: WMH, white matter hyperintensities; TBV, total brain volume; HCV, hippocampus volume; WMV, white matter volume; GMV, grey matter volume; COPD, chronic obstructive pulmonary disease; SBP, systolic blood pressure; DBP, diastolic blood pressure; FEV, forced expiratory volume; FVC, forced vital capacity; PEV, peaking expiratory volume; CKD, chronic kidney disease; BFP, body fat percentage; TFP, trunk fat percentage; HF, heart failure; CHD, chronic heart disease; AF, atrial fibrillation; BMR, basal metabolic rate; BMI, body mass index; HGS, hand grip strength.

**Figure S3. The results of restrict cubic spline analyses.**

**Figure S3a. The results of restrict cubic spline analyses for white matter hyperintensity.**

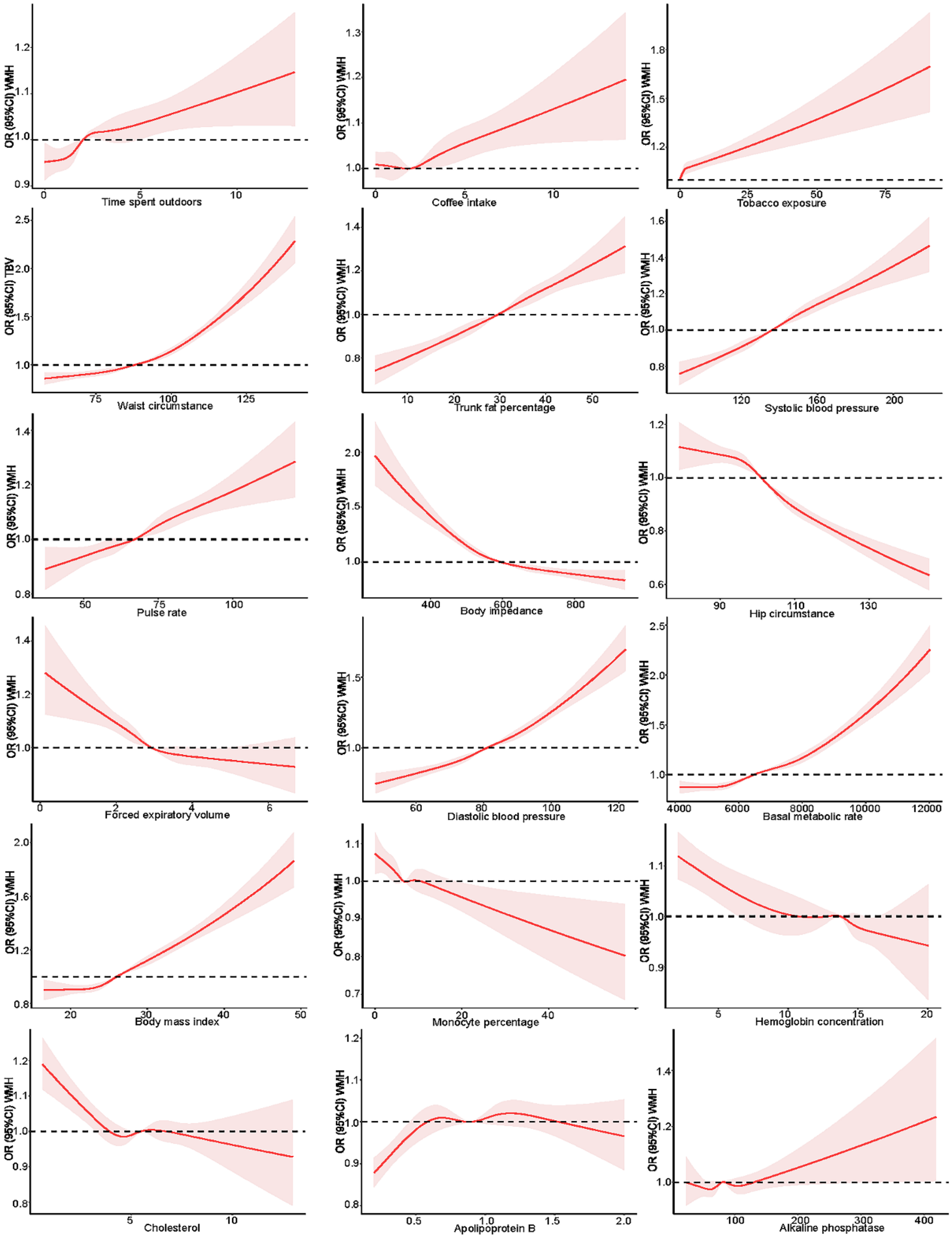

**Figure S3b. The results of restrict cubic spline analyses for hippocampus volume.**

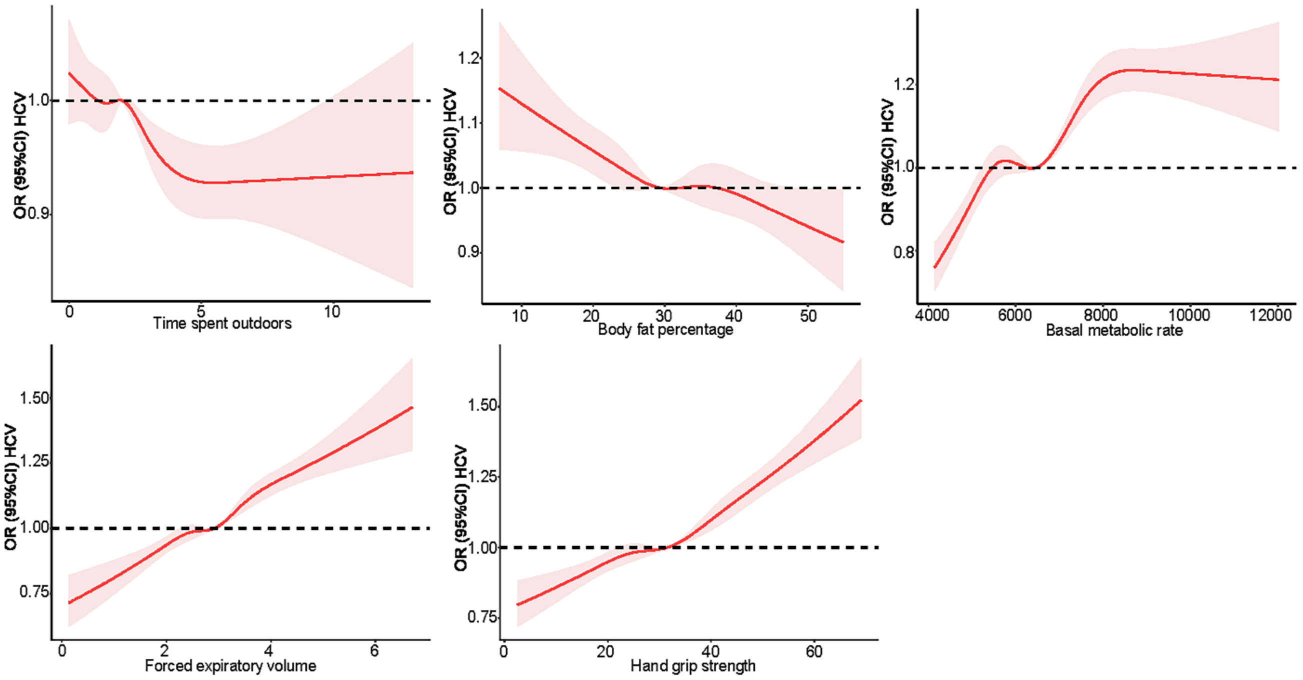


**Figure S3c. The results of restrict cubic spline analyses for total brain volume.**

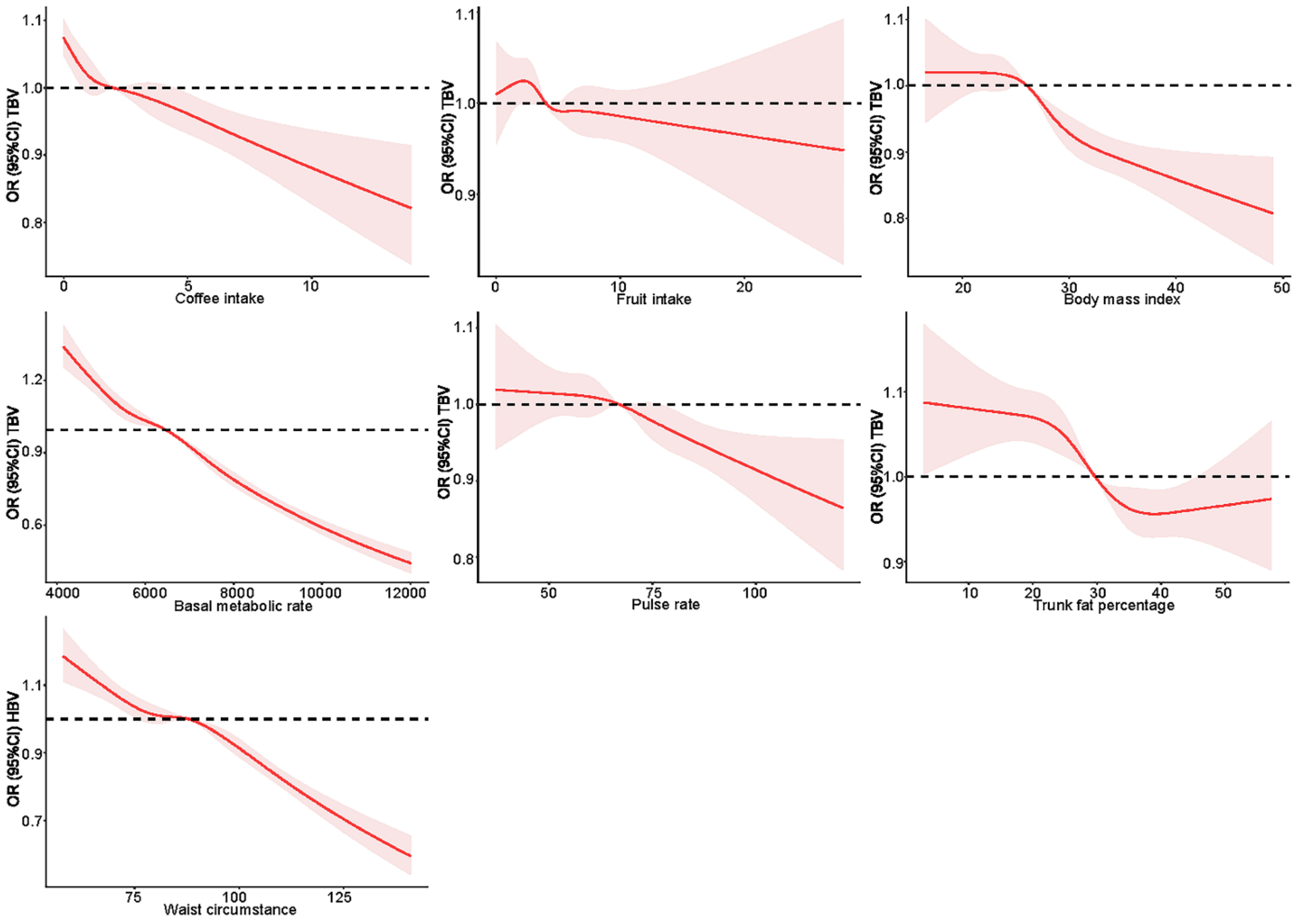

Figure S3d. The results of restrict cubic spline analyses for grey matter volume.

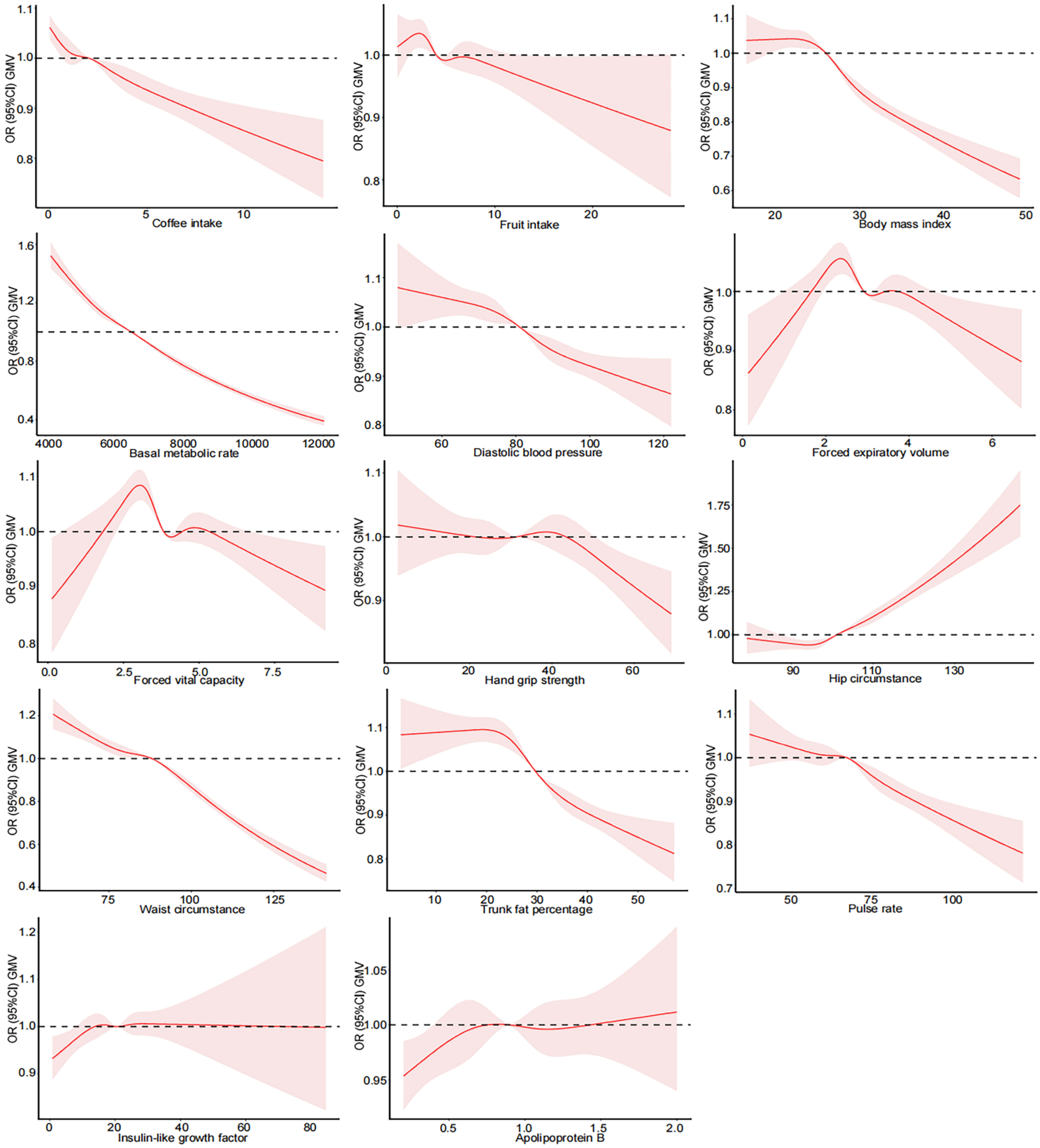

**Figure S3e. The results of restrict cubic spline analyses for white matter volume.**

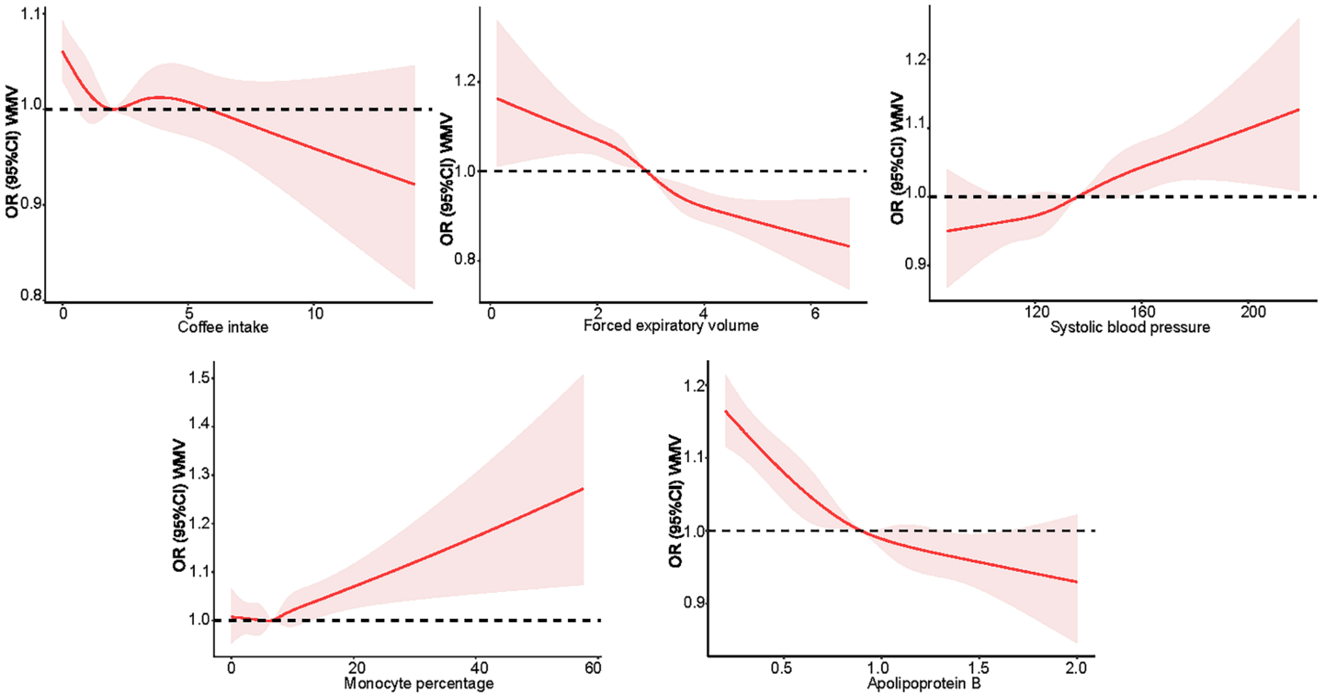

Supplement: Supplementary file 1 — Data S1. [file CNS-30-e70057-s001.pdf]
